# Mitochondrial genomes reveal recombination in the presumed asexual *Fusarium oxysporum* species complex

**DOI:** 10.1186/s12864-017-4116-5

**Published:** 2017-09-18

**Authors:** Balázs Brankovics, Peter van Dam, Martijn Rep, G. Sybren de Hoog, Theo A. J. van der Lee, Cees Waalwijk, Anne D. van Diepeningen

**Affiliations:** 1 Westerdijk Fungal Biodiversity Institute, Uppsalalaan 8, Utrecht, 3584CT The Netherlands; 2 Institute of Biodiversity and Ecosystem Dynamics, University of Amsterdam, Science Park 904, Amsterdam, 1098 XH The Netherlands; 3 Swammerdam Institute for Life Sciences, University of Amsterdam, Science Park 904, Amsterdam, 1098 XH The Netherlands; 4 Wageningen University and Research Centre, Droevendaalsesteeg 4, Wageningen, 6708 PB The Netherlands

**Keywords:** Comparative genomics, Mitochondrial genome, Mitochondrial recombination, Phylogenomics

## Abstract

**Background:**

The *Fusarium oxysporum* species complex (FOSC) contains several phylogenetic lineages. Phylogenetic studies identified two to three major clades within the FOSC. The mitochondrial sequences are highly informative phylogenetic markers, but have been mostly neglected due to technical difficulties.

**Results:**

A total of 61 complete mitogenomes of FOSC strains were *de novo* assembled and annotated. Length variations and intron patterns support the separation of three phylogenetic species. The variable region of the mitogenome that is typical for the genus *Fusarium* shows two new variants in the FOSC. The variant typical for *Fusarium* is found in members of all three clades, while variant 2 is found in clades 2 and 3 and variant 3 only in clade 2. The extended set of loci analyzed using a new implementation of the genealogical concordance species recognition method support the identification of three phylogenetic species within the FOSC. Comparative analysis of the mitogenomes in the FOSC revealed ongoing mitochondrial recombination within, but not between phylogenetic species.

**Conclusions:**

The recombination indicates the presence of a parasexual cycle in *F. oxysporum*. The obstacles hindering the usage of the mitogenomes are resolved by using next generation sequencing and selective genome assemblers, such as GRAbB. Complete mitogenome sequences offer a stable basis and reference point for phylogenetic and population genetic studies.

**Electronic supplementary material:**

The online version of this article (doi:10.1186/s12864-017-4116-5) contains supplementary material, which is available to authorized users.

## Background

Members of the *Fusarium oxysporum* species complex (FOSC) are important plant pathogens causing vascular wilts, rots and damping-off on a broad range of agronomically and horticulturally important crops [1, 2]. In addition, members of this species complex are also clinically important, causing infections in both human and animal hosts [3, 4]. Furthermore, despite the pathogenic potential of these fungi, not all strains are virulent. Putative non-pathogenic strains belonging to this group are used as biocontrol agents against pathogens [5].

The taxonomy of *Fusarium* has historically been based on the morphology of the asexual reproductive structures, leading to a broad definition of *F. oxysporum*. To capture intraspecific variability within the morphospecies, *formae speciales* (ff. spp.) were introduced based on the pathogenicity of the strains towards particular plant hosts [6]. *F. oxysporum* is closely related to the *F. fujikuroi* species complex that contains several heterothallic species. The genome of *F. oxysporum* resembles the genome of heterothallic species, both mating types can be found in populations, the mating type genes are expressed and introns are correctly spliced from the transcript [7]. However, since no sexual stage has been found for this species nor could it be induced under laboratory conditions, *F. oxysporum* is considered to be asexual [8, 9].

The use of molecular markers revealed that multiple ff. spp. have evolved polyphyletically [1, 10, 11]. Phylogenetic studies by O’Donnell et al. [8, 10] identified three major clades within the FOSC. However, later phylogenetic analysis conducted using genealogical concordance phylogenetic species recognition (GCPSR) based on eight loci supported the separation of only two phylogenetic species within the complex: one species corresponding to clade 1 and the other to the remaining clades [12].

The operational criteria for GCPSR for fungi were introduced in the theoretical paper of Taylor et al. [13], and first implemented as an operational framework using a two-step methodology by Dettman et al. [14]. After genetic isolation, two populations (or species) undergo the following stages: shared polymorphism (apparent polyphyly), loss of shared polymorphism (after fixation in one of the species) and reciprocal monophyly (after fixation in both species). According to GCPSR, the genetic isolation between populations (phylogenetic species) can be detected by a combination of genealogical concordance and non-discordance. Genealogical concordance is meant by the concordant reciprocal monophyly of multiple gene genealogies. Genealogical non-discordance means that no grouping supported by high support values for one of the genes is contradicted by another gene with the same level of support [13, 14].

Mitogenome sequences were used for resolving phylogenetic and evolutionary relationships between fungi at all taxonomic levels [15–17]. The advances in sequencing technologies and the reduction of costs involved, as well as, the development of tools to selectively assemble target genomic regions, like GRAbB, made it feasible to conduct complete mitochondrial genome (mitogenome) sequence analysis of a large number of strains [18].

The mitochondrial genomes of *Fusarium* spp. contain a set of fourteen “standard” mitochondrial polypeptide-encoding genes, two rRNA-encoding genes, *rnl* (mtLSU) and *rns* (mtSSU), and more than twenty tRNA-encoding genes [19]. The orientation and order of the genes are conserved within the genus, with the exception of some of the tRNA-encoding genes [17]. The mitochondrial genomes of *Fusarium* spp. contain variable numbers of introns, which causes significant size variation between the different species [17, 19]. One of the introns is located in the *rnl* gene and encodes a small ribosomal protein Rps3. This intron is conserved in the *Pezizomycotina* [20].

**Fig. 1:**
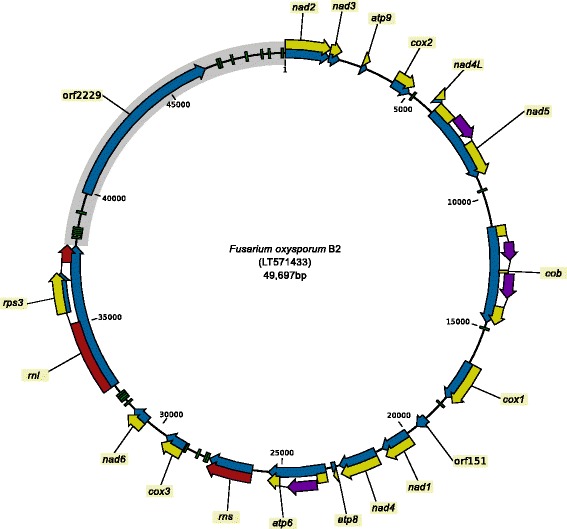
Mitochondrial genome of *Fusarium oxysporum* f. sp. *cubense* race 4 (strain B2; LT571433). Green blocks: tRNA coding genes, blue arrows: genes, yellow arrows: protein coding sequences, red arrows: rDNA coding sequence, purple arrows: intron encoded homing endonuclease genes, gray segment: large variable (LV) region with orf2229 (LV-uORF)

In addition to the genes with functional predictions, a large open reading frame (ORF) was found in all *Fusarium* spp. except in *F. oxysporum* (represented by strain F11) [17, 19, 21]. This large ORF was found in a region that is variable and contains several tRNA genes. For these reasons the region was referred to as large variable (LV) region and the large ORF with unknown function as LV-uORF [19] (orf2229 in Fig. 1). The mitogenome of the representative strain for *F. oxysporum* was sequenced using Sanger sequencing and primer walking [21]. This sequence did not contained the LV-uORF, and this is the only *F. oxysporum* mitogenome that was used for comparative studies so far [17, 19, 21]. Although several *F. oxysporum* strains have been sequenced using next generation sequencing (NGS) methods, there are only two strains for which the complete mitogenome was assembled. Both of these mitogenome sequences, GenBank accession no. KR952337 (unpublished) and LT571433 [18] (Fig. 1), contain the large variable region with the large variable ORF.

The goals of this study were (i) to prove that the mitochondrial genomes of a large number of strains can be analyzed, as we suggested in an earlier study [18], (ii) to present a detailed analysis of these mitochondrial genomes and (iii) to demonstrate how a detailed analysis of these genomes can contribute to our understanding of the biology of the given organism. To achieve these goals we present the following in this study. First, the phylogenetic relationships between 61 strains of the *F. oxysporum* species complex is analyzed. For this, we used a revised implementation of the GCPSR. Second, a detailed analysis of the genetic diversity of the mitochondrial genomes within the complex is given. Third, an interpretation of the mitogenome diversity in the light of the phylogeny is provided. Lastly, the biological implications of our results are discussed.

## Methods

### Fungal strains used and whole genome sequencing of the strains

Sixty-one *F. oxysporum* strains, two *F. proliferatum* strains and one *F. commune* strain were analyzed in this study (see Additional file 1). The *F. commune* strain (JCM 11502) was previously identified as *F. oxysporum*, but BLAST and Fusarium-MLST (http://www.westerdijkinstitute.nl/fusarium/) results showed that this strain belongs to *F. commune*.

The *F. oxysporum* f. sp. *cumini* strain F11, kindly provided by Dimitrios Tsirogiannis (Benaki Phytopathological Institute, Kifissia, Greece), was used for re-sequencing.

For the two *F. proliferatum* strains (ITEM2287 and ITEM2400), the two *F. oxysporum* f. sp. *dianthi* strains (Fod001 and Fod008) and *F. oxysporum* f. sp. *cumini* strain F11 shotgun libraries were made using the Illumina TruSeq nano DNA library prep kit, according to manufacturer’s protocols (Illumina). Each of the libraries was loaded as (part of) one lane of an Illumina paired-end flowcell for cluster generation using a cBot. Sequencing was done on an Illumina HiSeq2000 instrument using 101, 7, 101 flow cycles for forward, index and reverse reads respectively. De-multiplexing of resulting data was done using Casava 1.8 software. The sequencing data has been deposited into the European Nucleotide Archive (ENA) with the following accession numbers: PRJEB18591 (ITEM2287 and ITEM2400), PRJEB18594 (F11) and PRJEB18595 (Fod001 and Fod008) (see Additional file 1).

The remaining strains were sequenced by other research groups or as part of previous studies [22–24]. The sequencing reads for these strains were retrieved through NCBI’s Sequence Read Archive (see Additional file 1).

### Assembling sequences

The following nuclear protein coding genes were assembled from NGS data for all 64 strains: *γ*-actin (*act*), ATP citrate lyase (*acl1*), *β*-tubulin II (*tub2*), calmodulin (*cal*), nitrate reductase (*NIR*), phosphate permease (*PHO*), 60S ribosomal protein L10 (*rpl10a*), the largest and second largest subunit of DNA-dependent RNA polymerase II (*RPB1* and *RPB2*, respectively), translation elongation factor 1 *α* (*tef1a*), translation elongation factor 3 (*tef3*) and topoisomerase I (*top1*). Besides the nuclear protein coding genes, part of the mitochondrial SSU rRNA gene (mtSSU or *rns*), the complete nuclear rDNA repeat region (18S rRNA - ITS1 - 5.8S rRNA - ITS2 - 28S rRNA - IGS) and the complete mitochondrial genome were also assembled for all 64 strains.

The eight loci used by Laurence et al. [12] for genealogical concordance phylogenetic species recognition were only barcoding regions of the following genes: *acl1*, *tub2*, *cal*, *NIR*, *PHO*, *RPB1*, *RPB2* and *tef1a*. A set of eight complete protein coding genes were used in this study, which have been traditionally used as barcoding genes or have recently been suggested as such [25]: *act*, *tub2*, *cal*, *rpl10a*, *RPB2*, *tef1a*, *tef3* and *top1*.

All of the regions listed above were assembled from NGS reads using GRAbB (Genomic Region Assembly by Baiting; [18]) by specifying the appropriate reference sequence and employing SPAdes 3.6 [26, 27] as assembler. The assembled sequences have been uploaded to the European Nucleotide Archive under the following accession numbers: LT841199-LT841268 and LT905535-LT906358.

### Sequence annotation

The initial mitogenome annotations were done using MFannot (http://megasun.bch.umontreal.ca/cgi-bin/mfannot/mfannotInterface.pl) and were manually curated: annotation of tRNA genes was performed using tRNAscan-SE [28], annotation of protein-coding genes and the *rnl* gene was corrected by aligning intronless homologs to the genome. Intron encoded proteins were identified using NCBI’s ORF Finder (https://www.ncbi.nlm.nih.gov/orffinder/) and annotated using InterPro [29] and CD-Search [30]. The visualization of the annotated sequences was produced using CLC Sequence Viewer 7.7.1 (https://www.qiagenbioinformatics.com/products/clc-sequence-viewer/), the graphical output was subsequently manually adjusted.

### Sequence analysis

All sequence alignments were done using MUSCLE [31, 32]. The barcoding regions and genes were each aligned per locus. The alignment of the mitochondrial genome required a different approach. The mitochondrial genome sequences were split into two regions: the large variable region (between the *trnT(tgt)* and the *nad2* genes) and remaining part of the mitochondrial genome, which we refer to as the conserved part of the mitochondrial genome. Since the conserved part of the mitogenome and the variants of the large variable region were too long for MUSCLE to handle in a single run, we used the following approach. First, the given sequences were divided into non-overlapping homologous blocks (5000–7000 bp). Second, each of these blocks were aligned using MUSCLE. Finally, the individual aligned blocks were concatenated in the original order.

Sequence variability of each region was calculated by aligning the sequences, then the number of characters with multiple character states was calculated and divided by the total number of characters in the alignment. This step was done using fasta_variability from the fasta_tools package (https://github.com/b-brankovics/fasta_tools).

### Phylogenetic analysis

The most appropriate substitution evolution model was determined using jModelTest 2 [33] for each of the single locus data sets. Phylogenetic reconstruction has been conducted using MrBayes 3.2.5 [34]. The MCMC algorithm was run for 4,000,000 generations with four incrementally-heated chains, starting from random trees and sampling one out every 400 generations. Burn-in was set to a relative value, 0.25.

Majority-rule consensus (MRC) trees were calculated based on the trees generated by the MrBayes run using PAUP* 4.0a147 for Unix/Linux with “percent” set to 95 (corresponding to 0.95 posterior probability), using the two *F. proliferatum* strains and the *F. commune* strain as outgroups and rooting was set to “monophyl”.

### Genealogical concordance phylogenetic species recognition

Genealogical concordance phylogenetic species recognition (GCPSR) was implemented in two steps: (i) identifying independent evolutionary lineages (IELs) and (ii) exhaustive subdivision of strains into phylogenetic species. These were implemented using Perl scripts developed in house that are available at GitHub (https://github.com/b-brankovics/GCPSR). The GCPSR method applied in this study is a modified implementation of GCPSR *sensu* Dettman et al. [14]. The revised implementation of the GCPSR method is briefly described below (for a detailed description as well as recommendations see Additional file 2).

#### Identifying independent evolutionary lineages

Independent evolutionary lineages (IELs) have two criteria: concordance and non-discordance. In our analysis, a clade was considered concordant when it was supported by at least two single locus MRC phylogenies (for explanation on alternative settings, see Additional file 2). Clades “A” and “B” are discordant if *A*∩*B*≠*∅* (they have common elements) and neither one is a subset of the other. In our implementation, we used these two criteria in a sequential order: First, identifying concordant clades, then comparing concordant clades and removing those that were discordant with each other. The concordant clades obtained in this manner define an unambiguous tree topology. This tree can be visualized and the number of loci supporting each clade can be used as a support value for the given clade. This tree can be used for exhaustive subdivision.

#### Exhaustive subdivision and phylogenetic species recognition

After identifying IELs, the IELs that are supported by a large number of single locus genealogies (e.g. half of the loci), are considered as putative phylogenetic species, the rest of the IELs is removed (see Figure S2b in Additional file 2). Each isolate must be classified within a putative phylogenetic species. When an isolate is grouped within a given clade (putative phylogenetic species), then all subclades of the given clade are removed (see Figure S2c in Additional file 2). This is referred to as exhaustive subdivision, which ensures that all phylogenetic species are monophyletic and there are no paraphyletic species. The clades that are kept after this step are recognized as phylogenetic species.

## Results

### Phylogenetic analysis and GCPSR

**Fig. 2:**
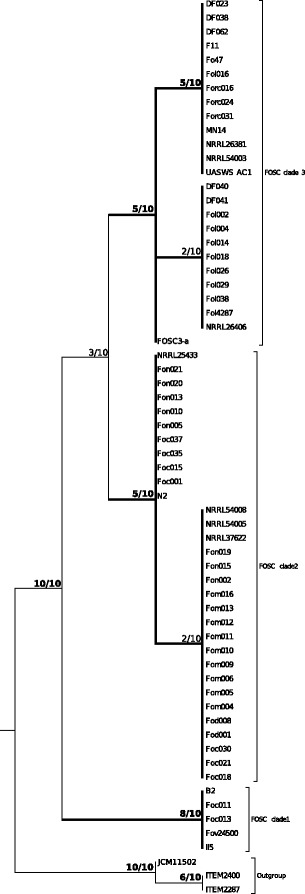
Genealogical concordance phylogenetic species recognition based on the 10 loci data set. The 10 loci used in the analysis were: *act*, *tub2*, *cal*, *tef1a*, *tef3*, *rpl10a*, *rpb2*, *top1*, rDNA repeat and the conserved part of the mitogenome. Only clades that were highly supported (BPP $\geqslant 0.95$) in at least two single locus phylogenies were included in the analysis. The three clades within the FOSC were recognized as phylogenetic species and shown in the tree. The support values indicate how many single locus phylogenies supported the given clade with BPP $\geqslant 0.95$ out of the 10 loci

Sixty-one strains belonging to *Fusarium oxysporum* species complex, one *F. commune* strain and two *F. proliferatum* strains have been analyzed in this study. Applying genealogical concordance phylogenetic species recognition (GCPSR) on the eight single copy nuclear (complete) protein coding genes (*act*, *cal*, *RPB2*, *rpl10a*, *tef1a*, *tef3*, *top1* and *tub2*) resulted in the recognition of three phylogenetic species within the FOSC. All three clades were present in at least half of the single locus phylogenies with high support (BPP $\geqslant 0.95$). Half of the single locus phylogenies still supported the three species when the rDNA repeat region and the conserved part of the mitogenome were added besides the eight genes (Fig. 2). The five loci that supported the recognition of all three clades with high support were among the six most variable loci included in the analysis (Table 2). The phylogeny of the conserved part of the mitogenome also supported the recognition of the three phylogenetic species that correspond to clades 1, 2 and 3 *sensu* O’Donnell et al. [10, 35].

In comparison, GCPSR based on the eight partial loci (*acl1*, *cal*, *NIR*, *PHO*, *RPB1*, *RPB2*, *tef1a* and *tub2*) used by Laurence et al. [12] had insufficient support to recognize the three phylogenetic species within the FOSC. Only *tef1a* showed high support for all three clades (data not shown).

### Mitogenomes

**Fig. 3:**
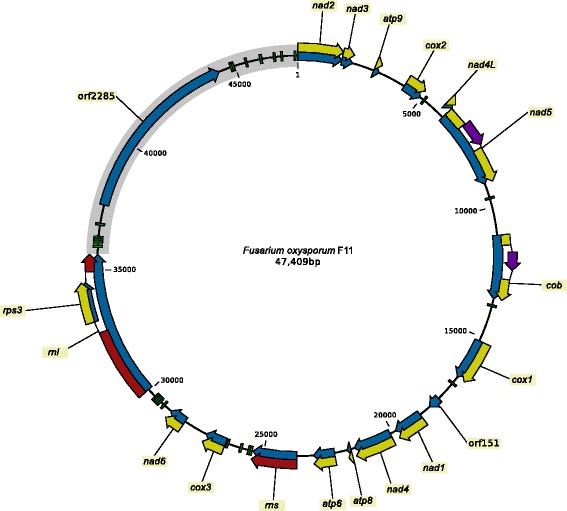
Mitochondrial genome of *Fusarium oxysporum* f. sp. *cumini* strain F11. Green blocks: tRNA coding genes, blue arrows: genes, yellow arrows: protein coding sequences, red arrows: rDNA coding sequence, purple arrows: intron encoded homing endonuclease genes, gray segment: large variable (LV) region with orf2285 (LV-uORF)

The mitogenomes of all 64 strains (61 FOSC and the 3 outgroup strains) were successfully assembled into single contigs each showing circular topology (Fig. 3). Some mitogenomes had identical sequences, in total there were 42 unique haplotypes identified for mitochondrial genome in this data set of 64 strains (Table 1).

**Table 1:** Mitochondrial genome lengths and intron presence. The strains of clades 2 and 3 are ordered based on the corrected mitochondrial genome length

Strain	Species	*forma specialis*	Clade	Introns				LV	mt length	Corrected^a^
				*nad5*	*cob*(1)	*cob*(2)	*atp6*	variant	(bp)	(bp)
Foc011	*F. oxysporum*	f.sp. *cucumerinum*	1	yes	yes	-	-	1	46476	33615
Foc013	*F. oxysporum*	f.sp. *cucumerinum*	1	yes	yes	-	-	1	46476	33615
Fov24500	*F. oxysporum*	f.sp. *vasinfectum*	1	yes	yes	yes	yes	1	48054	33727
B2	*F. oxysporum*	f.sp. *cubense*	1	yes	yes	yes	yes	1	49697	33419
II5	*F. oxysporum*	f.sp. *cubense*	1	yes	yes	yes	yes	1	49692	33414
Foc021	*F. oxysporum*	f.sp. *cucumerinum*	2	yes	-	-	-	2	50783	33449
Foc018	*F. oxysporum*	f.sp. *cucumerinum*	2	yes	-	-	-	2	50783	33449
Foc030	*F. oxysporum*	f.sp. *cucumerinum*	2	yes	-	-	-	2	50783	33449
NRRL25433	*F. oxysporum*	f.sp. *vasinfectum*	2	yes	-	-	-	1	44941	33458
N2	*F. oxysporum*	f.sp. *cubense*	2	yes	-	-	-	1	45639	33465
Foc035	*F. oxysporum*	f.sp. *cucumerinum*	2	yes	-	-	-	1	45706	33477
Fon002	*F. oxysporum*	f.sp. *niveum*	2	yes	-	-	-	1	45677	33477
Foc037	*F. oxysporum*	f.sp. *cucumerinum*	2	yes	-	-	-	1	45705	33479
Foc015	*F. oxysporum*	f.sp. *cucumerinum*	2	yes	-	-	-	1	45712	33486
Fom016	*F. oxysporum*	f.sp. *melonis*	2	yes	-	-	-	1	45538	33487
Fom013	*F. oxysporum*	f.sp. *melonis*	2	yes	-	-	-	1	45538	33487
Fom005	*F. oxysporum*	f.sp. *melonis*	2	yes	-	-	-	1	45538	33487
Fom012	*F. oxysporum*	f.sp. *melonis*	2	yes	-	-	-	1	45538	33487
NRRL54005	*F. oxysporum*	f.sp. *raphani*	2	yes	-	-	-	1	45536	33487
Fom004	*F. oxysporum*	f.sp. *melonis*	2	yes	-	-	-	1	45538	33487
Fom006	*F. oxysporum*	f.sp. *melonis*	2	yes	-	-	-	1	45538	33487
NRRL37622	*F. oxysporum*	f.sp. *pisi*	2	yes	-	-	-	3	39941	33490
Fom009	*F. oxysporum*	f.sp. *melonis*	2	yes	-	-	-	3	38736	33492
Fom011	*F. oxysporum*	f.sp. *melonis*	2	yes	-	-	-	3	38736	33492
Fom010	*F. oxysporum*	f.sp. *melonis*	2	yes	-	-	-	3	38736	33492
Fod008	*F. oxysporum*	f.sp. *dianthi*	2	yes	-	-	-	1	45694	33498
Fod001	*F. oxysporum*	f.sp. *dianthi*	2	yes	-	-	-	1	44947	33498
Foc001	*F. oxysporum*	f.sp. *cucumerinum*	2	yes	-	-	-	1	45741	33509
Fon015	*F. oxysporum*	f.sp. *niveum*	2	yes	-	-	-	1	45708	33514
Fon019	*F. oxysporum*	f.sp. *niveum*	2	yes	-	-	-	2	50087	33514
Fon020	*F. oxysporum*	f.sp. *niveum*	2	yes	-	-	-	1	45752	33526
Fon005	*F. oxysporum*	f.sp. *niveum*	2	yes	-	-	-	1	45752	33526
Fon010	*F. oxysporum*	f.sp. *niveum*	2	yes	-	-	-	1	45752	33526
Fon021	*F. oxysporum*	f.sp. *niveum*	2	yes	-	-	-	1	45752	33526
Fon013	*F. oxysporum*	f.sp. *niveum*	2	yes	-	-	-	2	50795	33527
NRRL54008	*F. oxysporum*	f.sp. *conglutinans*	2	yes	-	-	-	2	50023	33534
Forc031	*F. oxysporum*	f.sp. *radicis-cucumerinum*	3	yes	yes	-	-	1	47541	33667
DF023	*F. oxysporum*	f.sp. *lycopersici*	3	yes	yes	-	-	2	52324	33667
Fo47	*F. oxysporum*		3	yes	yes	-	-	1	47547	33667
Forc024	*F. oxysporum*	f.sp. *radicis-cucumerinum*	3	yes	yes	-	-	1	47541	33667
Forc016	*F. oxysporum*	f.sp. *radicis-cucumerinum*	3	yes	yes	-	-	1	47541	33667
NRRL54003	*F. oxysporum*	f.sp. *lycopersici*	3	yes	yes	-	-	1	47588	33674
UASWS AC1	*F. oxysporum*		3	yes	yes	-	-	1	47598	33682
DF038	*F. oxysporum*	f.sp. *lycopersici*	3	yes	yes	-	-	1	47562	33688
DF062	*F. oxysporum*	f.sp. *lycopersici*	3	yes	yes	-	-	1	47562	33688
Fol016	*F. oxysporum*	f.sp. *lycopersici*	3	yes	yes	-	-	1	47562	33688
NRRL26406	*F. oxysporum*	f.sp. *melonis*	3	yes	yes	-	-	1	47444	33688
Fol004	*F. oxysporum*	f.sp. *lycopersici*	3	yes	yes	-	-	2	52352	33694
Fol014	*F. oxysporum*	f.sp. *lycopersici*	3	yes	yes	-	-	2	52352	33694
Fol4287	*F. oxysporum*	f.sp. *lycopersici*	3	yes	yes	-	-	2	52352	33694
Fol038	*F. oxysporum*	f.sp. *lycopersici*	3	yes	yes	-	-	2	52352	33694
DF041	*F. oxysporum*	f.sp. *lycopersici*	3	yes	yes	-	-	2	52352	33694
Fol018	*F. oxysporum*	f.sp. *lycopersici*	3	yes	yes	-	-	2	52352	33694
Fol002	*F. oxysporum*	f.sp. *lycopersici*	3	yes	yes	-	-	2	52352	33694
Fol026	*F. oxysporum*	f.sp. *lycopersici*	3	yes	yes	-	-	2	52352	33694
DF040	*F. oxysporum*	f.sp. *lycopersici*	3	yes	yes	-	-	2	52352	33694
Fol029	*F. oxysporum*	f.sp. *lycopersici*	3	yes	yes	-	-	2	52352	33694
MN14	*F. oxysporum*		3	yes	yes	-	-	1	47573	33696
F11	*F. oxysporum*	f.sp. *cumini*	3	yes	yes	-	-	1	47409	33704
NRRL26381	*F. oxysporum*	f.sp. *radicis-lycopersici*	3	yes	yes	-	-	1	47263	33707
FOSC3-a	*F. oxysporum*		3	yes	yes	yes	-	2	53639	33727
JCM11502	*F. commune*		-	yes	-	-	-	1	47526	33565
ITEM2287	*F. proliferatum*		-	-	-	-	-	1	46555	33558
ITEM2400	*F. proliferatum*		-	-	-	-	-	1	46549	33549

^a^The length of the conserved part of the mitogenome after excluding the introns of protein coding genes

#### Intron presence

All of the strains analyzed contained an intron in the *rnl* gene (mtLSU) that is conserved in the *Pezizomycotina* and contains a gene coding for a ribosomal protein, Rps3. The two *F. proliferatum* mitogenomes contained no further introns. The *F. commune* mitogenome contained an intron in the *nad1* gene, which was not found in the other strains. The mitogenomes of the FOSC strains contained intron positions in the following protein coding genes: *atp6*, *cob* (this gene had two intron positions) and *nad5* (Table 1). The members of clade 2 could be differentiated from all other strains based on their intron patterns. Intron patterns showed almost no variation within clades 2 and 3, the only exception being strain FOSC3-a in clade 3. Clade 1 showed marked variation in intron pattern.

#### Genetic diversity of the conserved part of the mitogenome

The mitogenomes of *Fusarium spp.* contain a region that shows higher levels of variation than other parts of the mitogenome [19]. This region is referred to as the large variable (LV) region and it is located between *rnl* (mitochondrial LSU rRNA gene) and *nad2* (gray area in Fig. 3). In this paragraph, we present the analysis of the genetic diversity of the mitochondrial sequences located outside the LV region, the analysis of the LV region can be found in the next subsection.

Strains that belong to clades 2 and 3 had clearly different lengths of the conserved region of the mitogenome. This is partially due to the difference in intron patterns, but even after these introns were excluded, the two populations, clades 2 and 3, had significantly different lengths, 33492.32 ± 23.95 and 33688.68 ± 14.42, respectively (*p*-value <2.2∗10^−16^ according to two-sample t-test; Table 1 and Additional file 3). The significant difference between lengths of the conserved part of mitogenome suggests that these two populations have been genetically isolated. The variation observed in clade 1 was larger than in the two other clades. There were only 5 strains within clade 1, thus, further grouping of the strains based on mitogenome length within this clade would not produce statistically supported results.

#### Large variable region

Re-sequencing of *F. oxysporum* f. sp. *cumini* strain F11 revealed that the LV-uORF (orf2285) gene, typical for *Fusarium* spp., is present in the mitogenome of this strain at the expected position, within the large variable (LV) region located between *rnl* and *nad2* (Fig. 3).

**Fig. 4:**
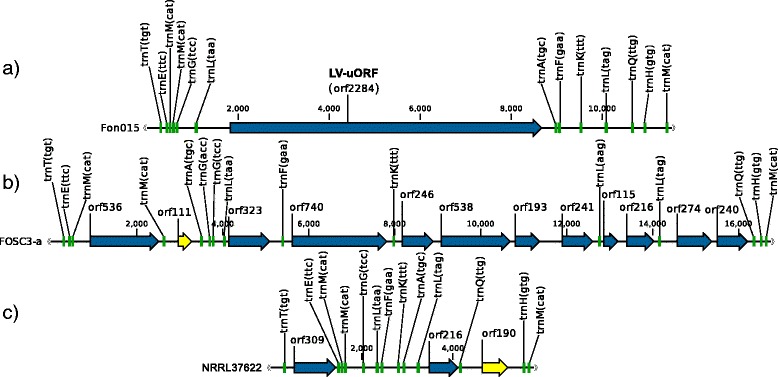
The three variants of the large variable region. **a** Variant 1 represented by *F. oxysporum* strain Fon015, **b** variant 2 represented by *F. oxysporum* strain FOSC3-a and **c** variant 3 represented by *F. oxysporum* strain NRRL37622. Green blocks: tRNA coding genes, blue arrows: ORFs, yellow arrows: ORFs that are not present in all representatives of the given variant

The LV region has three variants in the FOSC, which do not appear to be homologous (Fig. 4). Although the three variants contained tRNA genes with identical anticodon sequences in a similar order (Fig. 4), there is a high level of sequence variation between the three variants. BLAST was unable to identify homology between the tRNA genes of the different variants of the LV region. The variant that is typical for *Fusarium* spp., variant 1, was 13424 or 13428 bp long in the *F. proliferatum* strains, 12422 bp in the *F. commune* strain and 9833-12003 bp long in FOSC. This variant was present in all three major clades of the FOSC. Variant 2 of the LV region was 15816-16749 bp long and it was present only in clades 2 and 3 within the FOSC. Finally, variant 3 was 4570 or 5777 bp long and it was found only in clade 2.

#### Variant 1 of the LV region

This variant contained thirteen tRNA genes and the LV-uORF located between *trnL(taa)* and *trnA(tgc)* (Fig. 4). LV-uORF has no known function. Domain predictions returned low quality hits to a limited part of the protein sequence (see Additional file 4). Blast searches against the NCBI and UniProt databases returned hits against only *Fusarium* LV-uORF sequences. The GC-content of LV-uORF was higher than the average of that of conserved protein coding genes (both exonic and intronic regions). This higher GC-content was similar to that of the intergenic regions (see Additional file 4).

#### Variant 2 of the LV region

This variant contained fifteen tRNA genes, thirteen of which are also found in variant 1 and two additional tRNAs: *trnG(acc)* and *trnL(aag)*. This region contains eleven or twelve ORFs interleaved with the tRNA genes (Fig. 4). Most of the variant 2 sequences contain twelve ORFs, but strains Fon019 and NRRL54008 have lost the ORF found between the second copy of *trnM(cat)* and *trnA(tgc)*, due to a deletion.

Most of the ORFs have no functional prediction or returned no hits using BLAST. Four ORFs appeared to be homing endonuclease genes (HEGs) based on conserved domain matches: two ORFs, the one between the first and second *trnM(cat)* (orf536 in Fig. 4) and the one between *trnL(taa)* and *trnF(gaa)* (orf323 in Fig. 4), matched to LAGLIDADG endonucleases; the two ORFs between *trnL(tag)* and *trnQ(ttg)* gene (orf274 and orf240 in Fig. 4) matched to the GIY-YIG endonucleases.

#### Variant 3 of the LV region

This variant contained a set of thirteen tRNA genes that were also present in the other two variants. Strains Fom009, Fom010 and Fom011 contained an additional *trnQ(ttg)* gene. Besides the tRNA genes there were two ORFs present in all four strains containing this variant, while strain NRRL37622 contained an additional ORF (Fig. 4).

The two ORFs present in all strains with variant 3 of the LV region showed similarity to HEGs based on conserved domain search results, one belonging to the LAGLIDADG family and one to the GIY-YIG family. The putative LAGLIDADG HEG was located between *trnT(tgt)* and *trnE(ttc)* gene. The second ORF, between *trnL(tag)* and *trnQ(ttg)* gene, was a putative homolog of the GIY-YIG endonuclease gene upstream of *trnQ(ttg)* in variant 2, based on BLASTp results.

*F. oxysporum* f.sp. *pisi* strain NRRL37622 contains a third ORF, which returned partial hits to a hypothetical protein in *Rhizophagus irregularis* using BLASTp, and CD-Search (Conserved Domain Search [30]) showed similarity to a zinc-binding domain (zf-3CxxC). This ORF is located at a homologous position to the second *trnQ(ttg)* gene present in the other three strains possessing variant 3 of the large variable region (Fig. 4).

#### LV region phylogeny and conserved mitogenome phylogeny

**Fig. 5:**
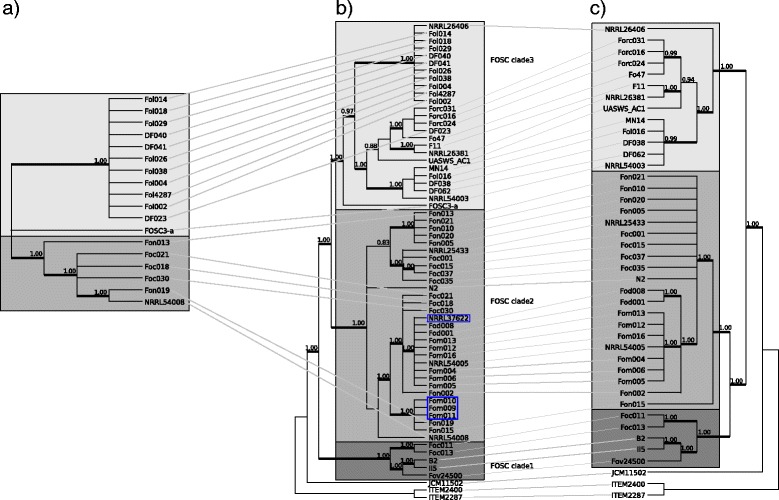
Tanglegram of the trees based on **a** the large variable region variant 2, **b** the conserved part of the mitogenome and **c** the variant 1 of the LV region, respectively. Clades with high Bayesian posterior probability (BPP) support are displayed with thicker branches. The support values are BPP values. The three phylogenetic clades identified within the FOSC are highlighted in different shades of gray. The strains that contain variant 3 are highlighted by blue boxes

In the phylogenetic tree based on variant 1 of the large variable region, strain Fov24500 grouped with members of clade 3, although this strain belonged to clade 1 based on other markers (data not shown). Sequence comparison between the LV-uORF of strain Fov24500 and other strains possessing the homologous region revealed that there is a large deletion in the copy found in Fov24500 (see Additional file 4). Since this deletion was unique to this strain, the phylogenetic reconstruction was also done after removing the LV-uORF region from the alignment of variant 1 sequences. The resulting tree and the phylogenetic tree of variant 2 are congruent with the phylogenetic tree based on the conserved part of the mitochondrial genome (presented as a tanglegram in Fig. 5 to highlight the co-evolution of these sequences). The topological incongruence is due to the differences in the resolution power of the regions. The only topological incongruence beyond the difference in resolution is the location of strain Fon015 within clade 2 of the FOSC. Fon015 was grouped with strains Fon019, Fom009, Fom010, Fom011 and NRRL54008 in the tree based on the conserved part of the mitogenome, while it appeared separate from all other clade 2 isolates in the tree based on variant 1 of the LV region.

#### Highly similar conserved region irrespective of different variants of the LV region

Sequence similarity of the conserved part of the mitochondrial genome was greater between members of the same clade than between strains that shared the same variant of the LV region, but belonging to different clades. The sequences of the conserved part of the mitogenome of *F. oxysporum* f. sp. *niveum* strains Fon015 and Fon019 from clade 2 were completely identical, despite the fact that they contain variants 1 and 2, respectively. A similar example was found in clade 3, where a high sequence similarity was observed between the conserved part of the mitogenome of the *F. oxysporum* f. sp. *melonis* strain NRRL 26406 and *F. oxysporum* f. sp. *lycopersici* strain FOL4287 (99.85%), despite the fact that they contain variants 1 and 2, respectively.

## Discussion

The theory of genealogical concordance phylogenetic species recognition (GCPSR) for fungi was introduced by Taylor et al. [13]. GCPSR consists of two steps: (i) identifying independent evolutionary lineages (IELs) based on genetic isolation estimated from multiple single locus phylogenies and (ii) exhaustive subdivision (classifying each isolate into clades substantiated by IELs). Previous implementations of GCPSR [12, 14] presented no programmatic tools for performing the analysis, required that balancing selection should not be influencing any of the loci in the analysis, identified IELs based on being at least concordant or non-discordant, and preformed exhaustive subdivision after superimposing the IELs on a maximum likelihood tree based on concatenated sequences. The last two characteristics make the method difficult to automate, which means that it is challenging to scale the method to a large number of loci. The scaling is further hindered by the requirement that loci should not be under balancing selection, which is difficult to detect before conducting phylogenetic comparisons.

We have employed a new strategy for GCPSR that uses sequential selection of clades: first identifying concordant clades, and subsequently removing the discordant clades from the selection. The programmatic implementation allows the user to set the minimum number of phylogenies required to recognize a given clade as concordant, which in combination with applying the two criteria sequentially makes the detection indifferent to some loci being under balancing selection. Our implementation of the GCPSR is scalable to any number of loci. Since the criteria for clade selection is applied sequentially and not in parallel, there can be no conflict left between the clades that are kept, and these clades already define a tree topology. The number of loci supporting a given clade can be used to display the support in the constructed tree. As a final selection, exhaustive subdivision can be used, by removing all subclades that would make a clade paraphylic. Employing exhaustive subdivision also ensures that no single strain can be recognized as unique species, thereby adhering to the genetic differentiation criterion.

Phylogenetic studies by O’Donnell et al. [8, 10] have identified three major clades within the *Fusarium oxysporum* species complex. Further phylogenetic analysis conducted using genealogical concordance phylogenetic species recognition based on eight loci supported the separation of two phylogenetic species within the complex: one species corresponding to clade 1 and the other to the remaining three clades [12].

In the current study, the eight loci used by Laurence et al. [12] were tested with our GCPSR approach. Only one locus, *tef1a*, contained sufficient phylogenetic information to support the grouping with sufficient Bayesian posterior support (BPP $\geqslant 0.95$) within the FOSC. In contrast, when the eight complete protein coding genes, the rDNA repeat region and the conserved part of the mitochondrial genome were used for the GCPSR analysis, three phylogenetic species were identified. All three species were highly supported in half of the single locus phylogenies. The three phylogenetic species correspond to clades 1, 2 and 3 *sensu* O’Donnell within the FOSC, and they were identified with low support (supported only by *tef1a*) by the eight loci analysis. The eight loci set did not contain sufficient phylogenetic information to separate the three species. The ten loci used for GCPSR contained more phylogenetic information, five out of the six most informative loci could resolve the three species with high support (BPP $\geqslant 0.95$, Table 2), the exception being the rDNA repeat region. Most of the variable sites in the rDNA repeat region were located in the intergenic spacer region (IGS), which is frequently the target of indel mutations [36]. Indel mutations make it difficult to estimate correctly which nucleotide characters are homologous, and this can lead to incorrect phylogenetic tree estimations. This could explain why the rDNA repeat sequence did not support the clustering supported by the other variable markers, despite the large number of parsimony informative sites.

**Table 2:** The variability of the individual loci used for the GCPSR based on the 10 loci data set. The loci are ordered based on the number of variable sites

Locus	Length	Conserved		PI^a^		The three clades
		Inter^b^	Intra^c^	Inter^b^	Intra^c^	were monophyletic
*rpl10a*	795	762	783	28	7	no
*cal*	979	920	963	52	8	no
*act*	1634	1572	1605	54	21	no
*tub2*	1671	1586	1634	78	27	no
*tef1a*	1776	1650	1711	99	43	yes
*tef3*	3588	3456	3532	111	43	yes
*top1*	3170	2981	3114	177	45	yes
*rpb2*	3907	3653	3812	213	61	yes
rDNA	7990	7437	7689	483	223	no
mt	40841	39121	40153	1501	464	yes

^a^Parsimony informative sites

^b^Outgroup + FOSC (clades 1, 2, 3)

^c^FOSC (clades 1, 2, 3)

The recognition of two phylogenetic species corresponding to clades 2 and 3 was further supported by the fact that the conserved part of their mitogenomes had significantly different lengths and their mitochondrial genomes had distinctly different intron patterns. This length difference was statistically significant even after excluding the introns of protein coding genes from the analysis. This marked difference suggests that the two populations have been genetically isolated. This genetic isolation could not be explained by the geographic origin of the strains.

The distribution of the variants of the large variable (LV) region of the mitogenome across the clades seems to contradict the recognition of the three phylogenetic species. However, the trees based on the variant regions and on the conserved part of the mitogenome are congruent. This demonstrates that three phylogenetic species are genetically isolated and differentiated from each other. It is likely that variants 1 and 2 of the LV region appeared in the common ancestor of clades 2 and 3, and they were maintained by recombination during the separation of the two lineages. This is supported by the fact that the two variants are present in both clades 2 and 3, and that the separation of clades 2 and 3 is supported by the phylogeny of both the LV region as well as the conserved part of the mitogenome. This hypothesis is also supported by the complete sequence identity of the conserved part of the mitogenome of *F. oxysporum* f. sp. *niveum* strains Fon015 and Fon019 from clade 2, despite the fact that these strains contain variants 1 and 2, respectively. A similar example in clade 3 is the high sequence similarity between the conserved part of the mitogenome of the *F. oxysporum* f. sp. *melonis* strain NRRL26406 and *F. oxysporum* f. sp. *lycopersici* strain FOL4287 (99.85%), while they contain variants 1 and 2 of the LV region, respectively.

In this study strain F11, which was used for previous comparative mitogenome studies [17, 19], was re-sequenced using next generation sequencing, and its mitogenome was assembled from the sequencing reads. The curated sequence contains the LV region, which is typical for *Fusarium* spp. [17, 19]. In addition, two introns were found that were absent from the original assembly [37].

Al-Reedy et al. [19] reported that the LV-uORF may represent a gene with unknown function and it is under purifying selection. Because the LV-uORF region has a higher GC content than the conserved protein encoding genes or the intron encoded genes and because its codon usage differs significantly from other mitochondrial genes, they suggested that the LV-uORF was acquired via horizontal gene transfer. However, examining the mitogenome sequence presented by Al-Reedy et al. [19] reveals that the GC contents of the LV-uORF and the intergenic regions are similar. So the GC content does not necessarily suggest horizontal gene transfer. Comparative genome analysis conducted in this study revealed that within the FOSC this region contains multiple indels as well as point mutations, which in some strains lead to fragmentation of the ORF into smaller ORFs (Additional file 4). The variability of this region as well as its GC content is similar to intergenic regions of the mitochondrial genome. All these facts combined suggest that the LV-uORF has lost its function within the FOSC or it never had a function within *Fusarium*. To understand whether the LV-uORF does have a function in other species complexes within *Fusarium* a study of the mitogenomes of these groups should be undertaken.

The analysis of the 61 strain data set revealed two additional variants of the LV region, both of which had no LV-uORF. Both contain at least thirteen tRNA genes containing the same anticodons that are present in variant 1 which is present in *F. proliferatum* as well. The different variants share only low sequence similarity and the order of the tRNA genes is not completely syntenic. Variant 1 is present in all three clades of the FOSC and also in other *Fusarium* spp. [17, 19], variant 2 is found in clades 2 and 3 within the FOSC, and variant 3 is confined to clade 2.

The origin of the two new variants of the LV region is unclear. They may have evolved from variant 1 by the insertion of mobile elements similar to mitochondrial introns, because the newly discovered variants contain ORFs that have domains matching those present in homing endonuclease genes (HEGs). HEGs are commonly associated with mitochondrial introns [38]. These may have initiated double strand breaks inside this region, which through homologous recombination repair could have led to the rearrangement of the tRNA genes, while still preserving some blocks of synteny.

*F. oxysporum* has been considered to be an asexual species. In asexual species, genetic exchange between strains is possible only between members of the same vegetative compatibility group (VCG). We found recombination of the mitochondrial genome, which indicates the presence of either a sexual or a parasexual cycle. The sexual cycle in haploid fungi involves the fusion of two haploid nuclei leading to a diploid nucleus which through meiosis results in haploid offspring. The parasexual cycle also begins by the fusion of two haploid nuclei, but recombination occurs by mitotic crossing over during multiplication of the diploid nucleus, and haploid cells emerge through vegetative haploidization instead of meiosis [39]. No sexual cycle could be induced in *F. oxysporum*, but the fusion of the nuclei of strains that were not members of the same VCG was shown [23, 40]. Genome analysis of *F. oxysporum* f. sp. *lycopersici* strain FOL4287 revealed that pathogenicity factors are located on accessory chromosomes and that transfer of these chromosomes to a non-pathogenic recipient conveyed pathogenicity to the recipient strain [23]. The proposed underlying mechanism for this chromosome transfer is that the nuclei of the two strains fuse, followed by selective loss of chromosomes from one of the fusion partners [40]. Genetic exchange may be restricted to members of the same phylogenetic species since anastomosis was observed between *F. oxysporum* f.sp. *lycopersici* Fol007 (clade 3) and *F. oxysporum* Fo47 (clade 3), between *F. oxysporum* f.sp. *lycopersici* Fol007 (clade 3) and *F. oxysporum* f.sp. *melonis* NRRL26406 (clade 3), but anastomosis could not be induced between *F. oxysporum* f.sp. *lycopersici* Fol007 (clade 3) and *F. oxysporum* f.sp. *cubense* NRRL25603 (clade 1) [23, 35]. These results demonstrates that recombination is possible between members of the same clade, but there is genetic isolation between the different clades.

## Conclusions

In this study a programmatic implementation of genealogical concordance phylogenetic species recognition (GCPSR) strategy was introduced, which allows the method to scale even to a large set of loci. It was shown to be robust in recognizing phylogenetic species. Our new GCPSR approach revealed three phylogenetic species within the *Fusarium oxysporum* species complex, which correspond to clades 1, 2 and 3 *sensu* O’Donnell et al. [10, 35]. We conclude that there has been genetic isolation between the different phylogenetic species leading to reciprocal monophyly of multiple loci. Thus, the phylogenetic species appear to define the limit of potential genetic exchange between members of the FOSC.

Both the recombination identified in the mitochondrial genome of the FOSC in our study and previous chromosome transfer studies [23, 40] indicate that there is a parasexual cycle in *F. oxysporum* and recombination is possible between members of the same phylogenetic species.

Mitochondrial genomes are highly informative for resolving phylogenetic relationships even between closely related species and populations. Complete mitochondrial genome sequences offer a stable basis and reference point for phylogenetic and population genetic studies. Our study demonstrates that a detailed comparative analysis of the mitogenome may offer new insights into the biology of the studied organism.

*F. oxysporum* strains contain the large variable region containing the LV-uORF that is not a functional gene. Furthermore, two new variants of the LV region were discovered within the FOSC. The distribution pattern and the sequence comparisons demonstrates that there has been mitochondrial recombination during the separation of clades 2 and 3. Clade 1 may contain more phylogenetic species. Extended sampling of this group may shed more light on the composition of the *Fusarium oxysporum* species complex.

## Additional files

Additional file 1 Strains analyzed and accession numbers of the NGS data used. (XLSX 9 kb)

Additional file 2 Scalable Genealogical Concordance Phylogenetic Species Recognition: Detailed description of the new implementation of the GCPSR method, including algorithm overview and recommended workflow. (PDF 420 kb)

Additional file 3 Boxplot of the length of the conserved part of the mitogenome of the three clades of the FOSC. (PDF 111 kb)

Additional file 4 Analysis of the LV-uORF region In depth comparative analysis of the LV-uORF region. (PDF 434 kb)

## Abbreviations

*acl1*: ATP citrate lyase
*act*: *γ-actin*
*atp6*: Mitochondrially encoded ATP synthase 6
*atp8*: Mitochondrially encoded ATP synthase 8
*atp9*: Mitochondrially encoded ATP synthase 9
BLAST: Basic Local Alignment Search Tool
BPP: Bayesian posterior probability
*cal*: Calmodulin
CD-Search: Conserved domain search
*cob*: Mitochondrially encoded cytochrome b
*cox1*: Mitochondrially encoded cytochrome c oxidase I
*cox2*: Mitochondrially encoded cytochrome c oxidase II
*cox3*: Mitochondrially encoded cytochrome c oxidase III
DNA: Deoxyribonucleic acid
ENA: European Nucleotide Archive
F: *Fusarium*
f. sp: *forma specialis*
ff. spp: *formae speciales*
FOSC: *Fusarium oxysporum* species complex
GCPSR: Genealogical concordance phylogenetic species recognition
GRAbB: Genomic Region Assembly by Baiting
GTR: General time reversible
HEG: Homing endonuclease
IEL: Independent evolutionary lineages
IGS: Intergenic spacer
ITS: Internal transcribed spacer
LV: Large variable
LV-uORF: Large variable open reading frame with unknown function
MCMC: Markov chain Monte Carlo
MRC: Majority-rule consensus
mt: Mitochondrial genome
*nad1*: Mitochondrially encoded NADH dehydrogenase 1
*nad2*: Mitochondrially encoded NADH dehydrogenase 2
*nad3*: Mitochondrially encoded NADH dehydrogenase 3
*nad4*: Mitochondrially encoded NADH dehydrogenase 4
*nad4L*: Mitochondrially encoded NADH dehydrogenase 4L
*nad5*: Mitochondrially encoded NADH dehydrogenase 5
*nad6*: Mitochondrially encoded NADH dehydrogenase 6
NCBI: National Center for Biotechnology Information
NGS: Next generation sequencing
*nir*: Nitrate reductase
ORF: Open reading frame
*pho*: Phosphate permease
rDNA: Ribosomal DNA
*rnl*: Mitochondrially encoded 16S RNA
*rns*: Mitochondrially encoded 12S RNA
*rpb1*: Largest subunit of DNA-dependent RNA polymerase II
*rpb2*: Second largest subunit of DNA-dependent RNA polymerase II
*rpl10a*: 60S ribosomal protein L10
*rps3*: Ribosomal protein S3
sp: Species (singular form)
spp: Species (plural form)
*tef1a*: Translation elongation factor 1 *α*
*tef3*: Translation elongation factor 3
*top1*: Topoisomerase I
tRNA: Transfer ribonucleic acid
*tub2*: *β-tubulin*
VCG: Vegetative compatibility group
