# Supplementary material for: Mitochondrial genomes reveal recombination in the presumed asexual Fusarium oxysporum species complex
Source: BMC Genomics. 2017 Sep 18;18:735. doi: 10.1186/s12864-017-4116-5 (PMC5604515; doi:10.1186/s12864-017-4116-5)
Supplement: Supplementary file 2 — Scalable Genealogical Concordance Phylogenetic Species Recognition: Detailed description of the new implementation of the GCPSR method, including algorithm overview and recommended workflow. (PDF 420 kb) [file 12864_2017_4116_MOESM2_ESM.pdf]

# Scalable Genealogical Concordance Phylogenetic Species Recognition

## 1 Algorithm overview

This section describes how the Perl scripts (available at GitHub: https://github.com/b-brankovics/GCPSR) implement the two steps of GCPSR method: (i) identifying independent evolutionary lineages (IELs) and (ii) exhaustive subdivision of strains into phylogenetic species. Both scripts have at least one parameter, the influence of these parameters on the outcome of the analysis, and the recommended interpretations of these results will be discussed in later sections. Each algorithmic step is marked by lowercase roman numerals for referencing and highlighting purposes (Figure S1).

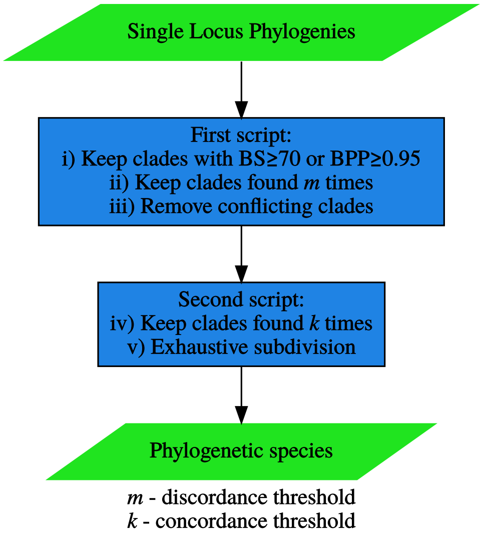


**Figure S1 Flowchart of the two step GCPSR method.** Green parallelograms are input and output files. Blue boxes are the scripts, roman numerals indicate the algorithmic steps. BS - bootstrap support value, BPP - Bayesian posterior probability value.

### 1.1 Identifying independent evolutionary lineages

The first script (concordance_non-discordance.pl) examines all the input trees (single-locus majority-rule phylogenies; in either newick or nexus format) and ($i$) saves all the clades with sufficient bootstrap (BS) or Bayesian posterior probability (BPP). The minimum sufficient support values is specified by the user when running the script. The occurrence of each of these clades is counted by the script, and ($ii$) only those clades are kept that are present in at least $m$ input trees. This

minimum number ($m$) is also specified by the user when running the script. Finally, ($iii$) the remaining clades are screened for discordance, and all clades that are in conflict with any other clades in the selection are removed. Clades "A" and "B" are discordant if $A \cap B \neq \emptyset$ (they have common elements) and neither one is a subset of the other. The concordant and non-discordant clades obtained in this manner define an unambiguous tree topology, which is printed as output by the script. The tree produced is in newick format, where the clade support values indicate how many of the input trees contained the given clade with sufficient support.

### 1.2 Exhaustive subdivision

The second script (exhaustive_subdivision.pl) takes the tree produced by the first script as input tree. The clades are read from the tree, and ($iv$) the clades with sufficient support ($\geq k$) are kept and the rest is discarded, the minimum support value (the number of single-locus majority-rule phylogenies containing the given clade with sufficient support; see step $i$) is specified by the user when running the script. Then ($v$) each of the strains present in the input tree are grouped into the least inclusive clade (clade with the fewest strains) containing that given strain, subsequently, all subclades of the given clade are removed (Figure S2). Clade "A" is a subclade of "B" if all the strains found in clade "A" are also present in clade "B". This last step ($v$) ensures that all clades are monophyletic. The final tree with only monophyletic clades is printed with clade support values indicating the number of single-locus majority-rule phylogenies containing the given clade with sufficient support (see step $i$).

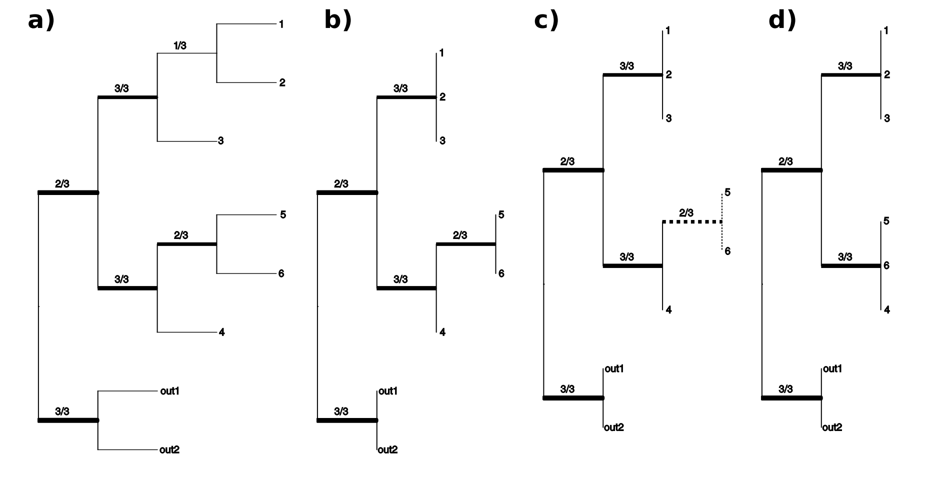


**Figure S2 Example for exhaustive subdivision with concordance threshold set to 2.** Support values indicate how many single-locus phylogenies support the clade out of 3 loci. Clades that have at least 2/3 support have thicker branches. **a)** Initial tree with all branches. **b)** After collapsing all branches with insufficient support. **c)** Strain "4" is grouped in clade "(4,5,6)", so its subclade, "(5,6)" has to be removed, although it has sufficient support (2/3). **d)** Final tree after subdivision.

## 2 Effect of parameters on the results

The first parameter is used in step $i$ by the first script, which will be referred to as minimum statistical support. This parameter defines the minimum support value (BS or BPP) a clade needs to have before it is kept for consideration for

concordance. The recommendation is to use a value which is considered significant for the given statistical method (BS≥70 and BPP≥0.95). Considering clades with significant statistical support helps to ensure the genetic differentiation criterion.

The second parameter is used in step *ii* by the first script, which will be referred to as discordance threshold ($m$). This parameter defines the minimum number of single-locus majority-rule phylogenies containing the given clade with at least minimum statistical support. The lower the discordance threshold is, the stricter the discordance analysis gets, since only concordant clades (clades kept after step *ii*) are tested for discordance. When the discordance threshold is set to 1, then a clade is discarded if any of the single-locus genealogies contradicts it. Setting a higher value for the discordance threshold means that clades supported by fewer single-locus genealogies than the threshold value will be ignored.

The final parameter is used in step *iv* by the second script, which will be referred to as concordance threshold ($k$). This parameter also defines the minimum number of single-locus majority-rule phylogenies containing the given clade with at least minimum statistical support, just as the concordance threshold. The difference between the role of the two parameters is that the discordance threshold influences the strictness of the discordance analysis, while the concordance threshold influences the strictness of phylogenetic species recognition. The higher the concordance threshold value is, the stricter the concordance criterion gets.

## 3 Recommended workflow

Earlier implementations of the GCPSR used independently evolving, phylogenetically informative loci that were considered as reliable phylogenetic markers.—In this document, phylogenetic information (as in phylogenetically informative) refers to the information a given locus contains that can be used for building trees. A phylogenetically informative locus is a locus that contains a significant amount of parsimony informative sites.—Not reliable phylogenetic markers would be loci that are under balancing selection or loci that cannot be aligned unambiguously, since improperly aligned loci do not produce reliable tree predictions. Balancing selection can maintain allelic polymorphism even through speciation.

Applying the GCPSR to large numbers of loci, means that for most of the loci we do not know whether they violated these assumptions before running the analysis. For this reason, the recommendation is to divide the analysis into different phases: identifying general evolutionary trends (clusters), investigating discordant genealogies and resolving phylogenetic species based on refined set of loci.

### 3.1 Phase 1: Identifying general evolutionary trends

The first question is whether there are multiple lineages that could potentially be recognized as phylogenetic species based on the data set. The process used in this phase is similar to calculating trees based on concatenated alignments, the goal of this part of the analysis is not to reveal actual phylogenetic species, but to identify clustering that may prove to be phylogenetic species in phase 3. In this phase of the analysis a relatively large discordance threshold ($m$; ideal this would be half of the loci used, but this depends on how informative[*] the loci are) should be used, so that

[*] How rich the locus is in parsimony informative sites.

a small number of conflicting loci does not mask the general pattern (clustering) indicated by the majority of loci. Potential phylogenetic species can be identified using the second script on the output of the first script. If potential phylogenetic species are found in the output tree, then individual conflicting genealogies have to be investigated (phase 2).

### 3.2 Phase 2: Investigating discordant genealogies

It has to be tested if any of the single-locus majority-rule phylogenies are discordant with the result of phase 1. This can be done by rerunning the scripts with discordant threshold ($k$) set to 1. If it produces the same result as phase 1, then only thing left is to resolve the phylogenetic species (phase 3). Otherwise, the loci that produce clades that are in conflict with the clades produced by the majority of the loci have to be identified and investigated more closely.

Discordant genealogies can be identified by using a third script (find_conflicting_tree.pl) that can compare the topology of each locus to the topology of the tree obtained in phase 1. All conflicting loci have to be examined separately, and it has to be investigated whether they should be considered as acceptable markers for GCPSR. For instance, loci that are under balancing selection should not be used for GCPSR, as the gene genealogies are not expected to reflect species genealogies. Another example could be paralogs that were thought to be orthologs, which also may result in genealogies that do not reflect the species phylogeny. All loci that cannot be considered as reliable markers for species phylogeny should be removed from further analysis.

The discordance between different loci can be the result of intraspecies recombination. According to Taylor *et al.* [13]: “The transition from concordance among branches to incongruity among branches can be used to diagnose species.” Within a single phylogenetic species the loci are subject to recombination, while after the separation of species, lineage sorting results in reciprocal monophyly at multiple loci.

### 3.3 Phase 3: Resolving phylogenetic species

After eliminating all the loci that cannot be considered as reliable markers for species phylogeny, the two step GCPSR can be rerun with minimum statistical support set to significant level (BS$\geq$70 or BPP$\geq$0.95), discordance threshold ($m$) set to 1[†], and concordance threshold ($k$) set sufficiently high (the proper concordance threshold is difficult to be defined). Following Dettman *et al.* [14], it should be the majority of the loci ($n/2$ or $n/2+1$, where $n$ is the number of loci used for the analysis), but they were using markers that were known to be informative[*] for the given taxon. When applying the method to a large number of loci mined from genomes, there is a chance that a large portion of the loci will not contain sufficient

† Using $m=1$, may prove to be overly conservative: the analysis is, basically, the same as the non-discordance analysis of the GCPSR method of Dettman *et al.* [14]. An alternative option could be using $m=2$; which would ignore groupings supported by only one single-locus phylogeny. The reasoning behind using $m=2$ is that some loci are probably contradicting the general trend in the genome, but this does not necessarily suggest that the two groups are not genetically isolated. However, multiple ($>1$) single-locus phylogenies showing the same grouping should be taken as a possibly significant deviation from the genetically isolated population hypothesis.

* How rich the locus is in parsimony informative sites.

phylogenetic information to separate the phylogenetic species. The concordance threshold could be adjusted so that it corresponds to $p/2$ or $p/2+1$, where $p$ is the number of phylogenetically informative[*] loci.

## 4 How to implement GCPSR *sensu* Dettman *et al.* using the two scripts

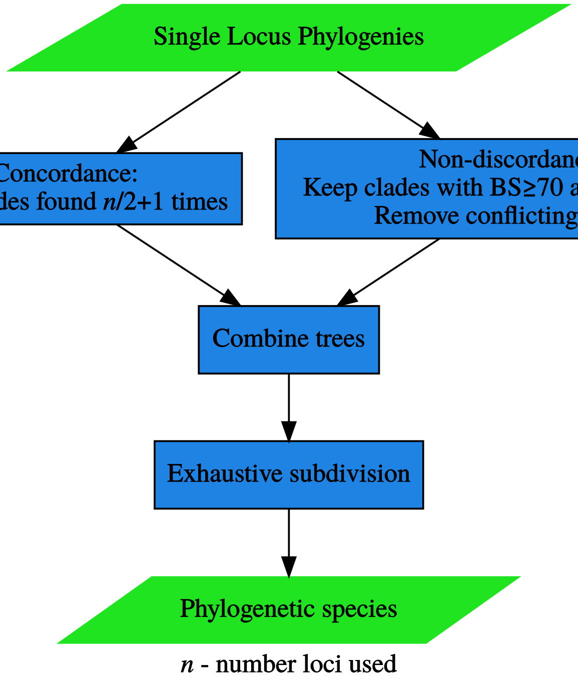


**Figure S3 Flowchart of the GCPSR method *sensu* Dettman *et al.*.** Green parallelograms are input and output files. Blue boxes are the scripts, roman numerals indicate the algorithmic steps. BS - bootstrap support value, BPP - Bayesian posterior probability value.

Dettman *et al.* [14] applied the two criteria, concordance and non-discordance, separately to recognize independent evolutionary lineages (Figure S3). The requirement for concordance according to their framework is that a clade has to be present in the majority of single-locus majority-rule consensus phylogenies. To run this analysis, the first script has to be run with minimum statistical support set to 0 and discordance threshold $(m)$ set to $n/2$ or $n/2+1$, where $n$ is the number of loci used for the analysis. The requirement for non-discordance is that a clade is well supported in at least one single-locus genealogy and not contradicted in any other single-locus genealogy at the same level of support. To achieve the same result using the first script, it has to be run with minimum statistical support set to 70 for BS and 0.95 for BPP, and discordance threshold $(m)$ set to 1.

In the original description of the method, it is not discussed how conflicting results produced by the two analyses should be handled. Assuming that both analyses produce equally valuable independent evolutionary lineages, the two trees produced can be combined by using the first script with minimum statistical support set to 0 and discordance threshold $(m)$ set to 1. The final step, phylogenetic species

[*] How rich the locus is in parsimony informative sites.

recognition, can be done by running the second script on the combined tree with concordance threshold ($k$) set to 1.
