# Supplementary material for: Mitochondrial genomes reveal recombination in the presumed asexual Fusarium oxysporum species complex
Source: BMC Genomics. 2017 Sep 18;18:735. doi: 10.1186/s12864-017-4116-5 (PMC5604515; doi:10.1186/s12864-017-4116-5)
Supplement: Supplementary file 3 — Boxplot of the length of the conserved part of the mitogenome of the three clades of the FOSC. (PDF 111 kb) [file 12864_2017_4116_MOESM3_ESM.pdf]

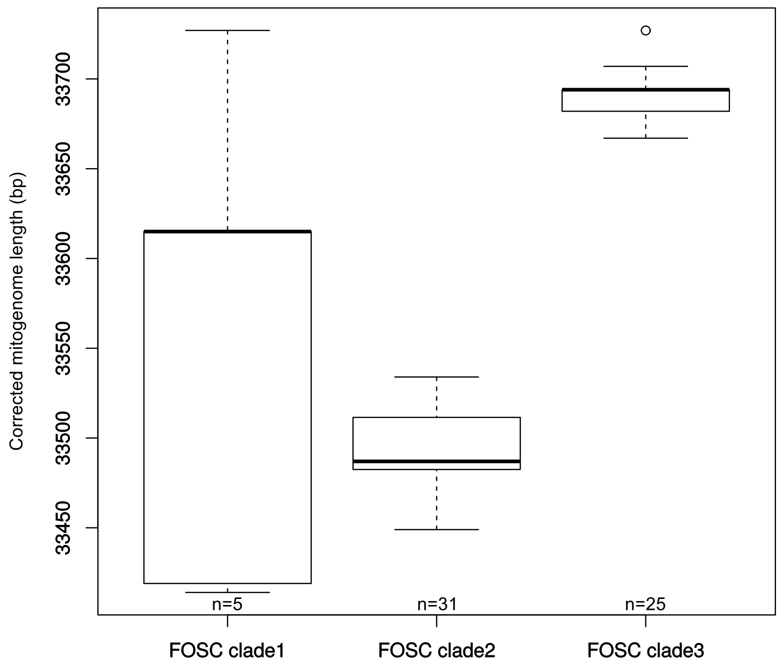

Figure S4 Boxplot of the length of the conserved part of the mitogenome of the three clades of the FOSC. The length used for the plot is the length of the conserved region of the mitogenome after excluding the introns of the protein coding genes.
