# Supplementary material for: Mitochondrial genomes reveal recombination in the presumed asexual Fusarium oxysporum species complex
Source: BMC Genomics. 2017 Sep 18;18:735. doi: 10.1186/s12864-017-4116-5 (PMC5604515; doi:10.1186/s12864-017-4116-5)
Supplement: Supplementary file 4 — Analysis of the LV-uORF region In depth comparative analysis of the LV-uORF region. (PDF 434 kb) [file 12864_2017_4116_MOESM4_ESM.pdf]

# Analysis of the LV-uORF region

## 1 LV-uORF variability similar to intergenic variability

The intronic regions were more variable than the intergenic regions (71.74 % and 39.87 %, respectively), when indel mutations were also included in the calculation of variable sites (Table S2). To estimate the effect of point mutations, the variability measurements were also calculated by ignoring characters with gap character state. In this case, the intronic sequences showed greater conservation than the intergenic regions (variability: 1.50 % and 9.35 %, respectively; Table S2).

**Table S2 Genetic variability of the conserved region of the mitogenomes.**

| Species | Region | Length | Variable | PI | Variable (%) | PI (%) | Variable[1] | PI[1] | Variable (%)[1] | PI (%)[1] |
|---|---|---|---|---|---|---|---|---|---|---|
| All[2] | Coding | 19821 | 582 | 538 | 2.94 | 2.71 | 314 | 270 | 1.58 | 1.36 |
| All[2] | Intron | 7142 | 5124 | 5075 | 71.74 | 71.06 | 107 | 63 | 1.50 | 0.88 |
| All[2] | Intergenic | 13888 | 5537 | 5329 | 39.87 | 38.37 | 1299 | 1168 | 9.35 | 8.41 |
| FOSC | Coding | 19821 | 175 | 129 | 0.88 | 0.65 | 146 | 100 | 0.74 | 0.50 |
| FOSC | Intron | 7142 | 3927 | 3871 | 54.98 | 54.20 | 83 | 39 | 1.16 | 0.55 |
| FOSC | Intergenic | 13888 | 1630 | 1358 | 11.74 | 9.78 | 459 | 325 | 3.31 | 2.34 |

PI: parsimony informative site
[1] Characters with gap character state were ignored while calculating these values.
[2] Outgroup + FOSC

The variability of the LV-uORF region within variant 1 of the large variable region was similar to the variability of the intergenic region inside this idiomorph (54.06 % and 48.75 %, respectively; Table S3), especially when the gap character state was ignored (10.30 % and 9.78 %, respectively; Table S3). The intergenic region inside the LV region was more variable than in the conserved part of the mitogenome (54.06 % and 39.87 %, respectively; Table S2 and S3). The difference between the two was less apparent when the gap character state was ignored for the analysis (9.35 % and 10.30 %, respectively; Table S2 and S3).

**Table S3 Genetic variability of variant 1 of the large variable region.**

| Species | Region | Length | Variable | PI | Variable (%) | PI (%) | Variable[1] | PI[1] | Variable (%)[1] | PI (%)[1] |
|---|---|---|---|---|---|---|---|---|---|---|
| All[2] | LV-uORF | 7508 | 3660 | 2401 | 48.75 | 31.98 | 734 | 657 | 9.78 | 8.75 |
| All[2] | Intergenic | 5503 | 2975 | 2783 | 54.06 | 50.57 | 567 | 483 | 10.30 | 8.78 |
| All[2] | Not tRNA | 13011 | 6635 | 5184 | 51.00 | 39.84 | 1301 | 1140 | 10.00 | 8.76 |
| All[2] | tRNA | 888 | 2 | 2 | 0.23 | 0.23 | 2 | 2 | 0.23 | 0.23 |
| FOSC | LV-uORF | 7508 | 2765 | 1311 | 36.83 | 17.46 | 352 | 263 | 4.69 | 3.50 |
| FOSC | Intergenic | 5503 | 1174 | 960 | 21.33 | 17.45 | 233 | 144 | 4.23 | 2.62 |
| FOSC | Not tRNA | 13011 | 3939 | 2271 | 30.27 | 17.45 | 585 | 407 | 4.50 | 3.13 |
| FOSC | tRNA | 888 | 1 | 1 | 0.11 | 0.11 | 1 | 1 | 0.11 | 0.11 |

PI: parsimony informative site
[1] Characters with gap character state were ignored while calculating these values.
[2] Outgroup + FOSC

## 2 Fragmentation of LV-uORF

Some of the strains possessing this idiomorph contain mutations that fragment the ORF into multiple ORFs (Figure S5). The large ORF region of Foc011 and Foc013 contain two stop codons that fragment the ORF into three ORFs. The first split is

the result of a frameshift mutation (inside the codon of the 90th aa). The second mutation is a point mutation leading to an early stop codon, the same mutation is found in Fom004, where the ORF is split into two ORFs. The N2 strain contains an insertion that contains a stop codon and a start codon.

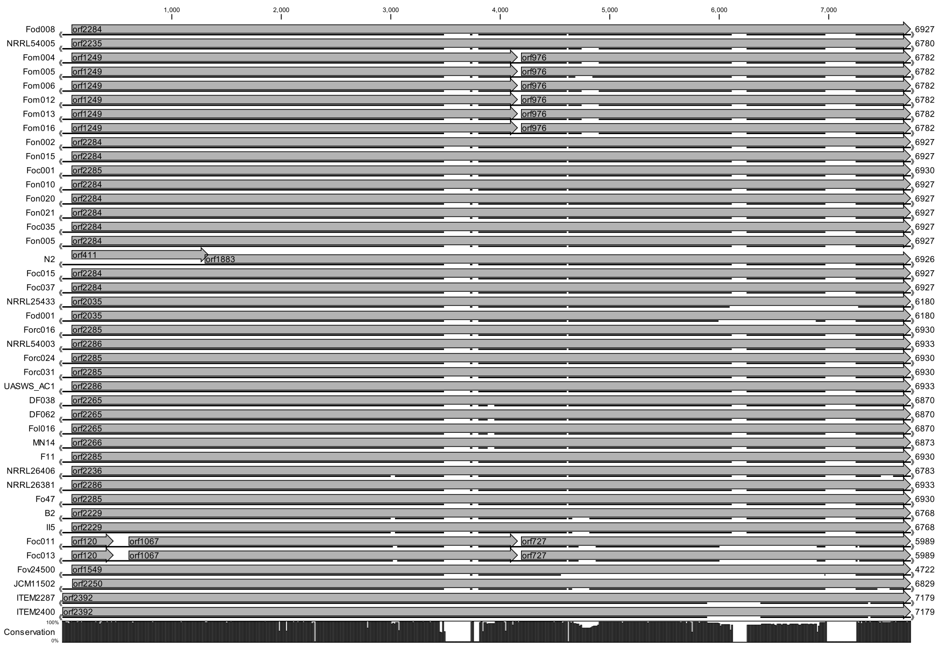


**Figure S5 DNA alignment of the LV-uORF region inside variant 1 of the large variable region.** Black lines indicate DNA sequences in the alignment, yellow arrows indicate ORFs, numbers at the end of the rows display the length of the region in the given strain and the conservation row depicts the level of conservation for the different regions.

## 3 GC-content of LV-uORF

The GC content of the conserved protein coding genes was 26.95% and that of the intronic sequences was 25.64%. These were significantly lower than that of the LV-uORF region, which was 34.91%. The GC content of the intergenic regions in the conserved part of the mitogenome (34.23%) and in the variant 1 of the LV region (36.28%) were more similar to that of the LV-uORF than that of the protein coding genes.

## 4 Functional prediction of LV-uORF

The LV-uORF has no known function. Annotation of the LV-uORF of *F. oxysporum* strain F11 identified possible transmembrane regions within the first 400 aa of the 2248 aa long protein, the rest of the protein sequence had no domain hits (Fig. S6). CD-Search has returned two partial hits. The first hit was between part of the region where InterPro predicted transmembrane regions and part of the conserved domain of DUF2070 (pfam09843) protein (Fig. S6). The DFU2070 is a family of Archeal 7-TM proteins. The hit covered 140 aa out of the 560 aa of the conserved domain. The second hit was between the central part of the LV-uORF and part of the conserved domain of SURF6 (pfam04935) (Fig. S6). The surfeit locus protein

SURF-6 is shown to be a component of the nucleolar matrix and has a strong binding capacity for nucleic acids. The hit covered 80 aa out of the 162 aa of the conserved domain. Blast searches against the NCBI and UniProt database returned hits against only *Fusraium* LV-uORF sequences.

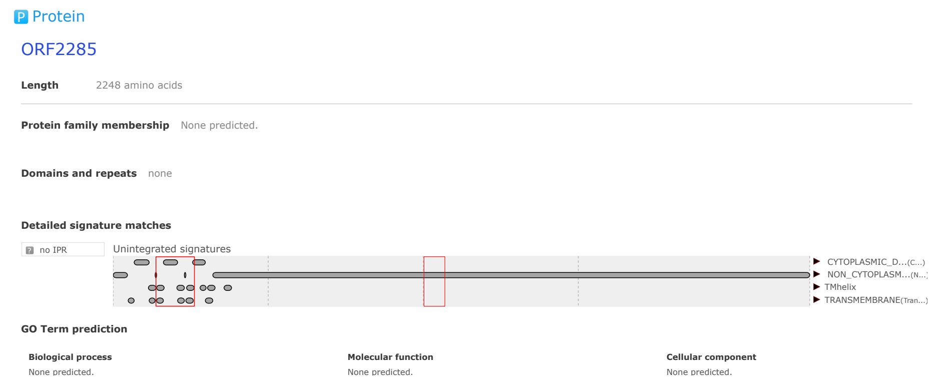


**Figure S6 InterPro functional prediction of LV-uORF (orf2248).** Red boxes indicate the position of CD-Search hits.
